# A re-evaluation of the peak *P*–*T* conditions of eclogite-facies metamorphism of the Paleozoic Acatlán Complex (Mexico) reveals deeper subduction

**DOI:** 10.1038/s41598-022-25992-8

**Published:** 2022-12-10

**Authors:** D. Hernández-Uribe

**Affiliations:** Department of Earth and Environmental Sciences, University of Illinois Chicago, Chicago, IL 60607 USA

**Keywords:** Petrology, Tectonics

## Abstract

Eclogites in the Acatlán Complex, southern Mexico, record the subduction history of the complex. Previous studies indicate that the proto-Acatlán Complex reached < 50 km depth during subduction. Yet, a recent study reported higher pressures for a single eclogite, questioning the maximum depth reached by the complex during subduction. In this work, I re-calculate eclogite pressure and temperature (*P*–*T*) conditions using thermobarometric methods applicable to eclogite-facies mafic rocks to a set of eclogites cropping out throughout the high-pressure belt of the Acatlán Complex—the Piaxtla Suite. I find that Acatlán eclogites record substantially—and systematically—greater pressures than previously reported. Calculations show that eclogites from the central part of the Piaxtla Suite (in the Piaxtla area) record consistent pressures of ~ 2.0 GPa and temperatures ranging between 460 and 675 °C. Eclogites from the northern part of the Piaxtla Suite (Mimilulco and Santa Cruz Organal areas) lack phengite, thus pressures were not calculated; temperatures calculated for these rocks at a fixed pressure (2.0 GPa) yield contrasting temperatures (511 °C and 870 °C, respectively). Mimilulco eclogite likely records similar pressures (~ 2.0 GPa) to other Piaxtla eclogites, whereas the pressures of Santa Cruz Organal eclogites might have been different, and likely experiencing a different thermal history compared to the rest of the eclogites from the Piaxtla Suite. Overall, these results indicate that the Acatlán Complex subducted to greater depths than previously thought implying a faster burial—exhumation cycle of the proto-Acatlán Complex.

## Introduction

Orogenic eclogites record key evidence of subduction-related processes and serve as important markers to identify paleo-subduction zones^1–3^. This type of eclogites occurs in two different convergent-margin regimes, namely the Pacific- and collision-type orogens. Pacific-type eclogites form along colder geotherms compared to collision-type eclogites, reach slightly lower peak pressures (2.0–2.3 GPa) than collision-type eclogites (> 2.3 GPa), and exhume at different rates than collision-type eclogites^3,4^. Therefore, constraining the pressure and temperature (*P–T*) evolution of eclogites is crucial for characterizing orogens, and specifically, for quantifying their burial—exhumation cycle.

**Figure 1:**
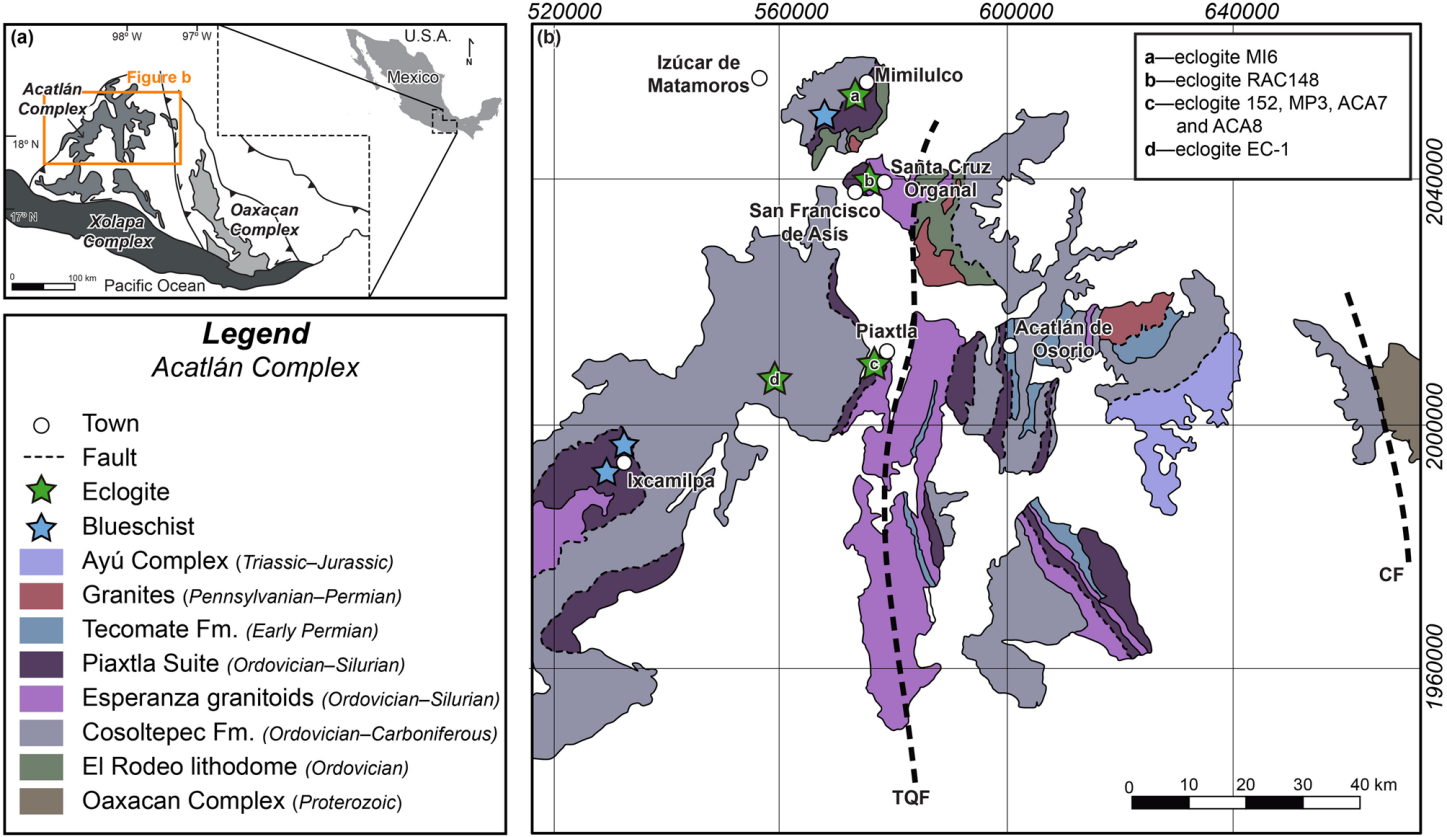
(**a**) Tectonostratigraphic terranes of southern Mexico. (**b**) Geological map of the Acatlán Complex. The green stars represent eclogite localities with the Piaxtla Suite (approximate locations). 152—Ortega-Gutiérrez^7^; MI6 and MP3—Meza-Figueroa et al.^8^; ACA7, ACA8, and RAC148—Vega-Granillo et al.^10^; EC-1—Hernández-Uribe et al.^6^; TQF—Tetla-Quicayán Fault; CF—Caltepec Fault. Modified from Hernández-Uribe et al.^6^.

The Acatlán Complex, southeastern Mexico (Fig. 1), exposes Paleozoic metamorphic rocks of various grades, including high-pressure (HP) lithologies such as chloritoid–rutile–phengite micaschists, garnet–rutile–phengite orthogneisses, garnet–epidote blueschists, and amphibole-phengite eclogites^5^. Eclogites in the Acatlán Complex represent the deepest subducted portions of the complex. A recent study^6^ reported maximum subduction depths of ~ 70 km—i.e. ~ 22–38 km higher than previously suggested for the Complex^7–12^, questioning accepted geodynamic models and exhumation rates for the Acatlán Complex^13,14^.

In this contribution, I re-evaluate the eclogite-facies metamorphism in the Acatlán Complex. For this, I calculated eclogite *P–T* conditions from different localities within the Acatlán Complex to evaluate whether the complex indeed experienced deeper subduction than previously thought or that this finding represents a single deep exposure within the Acatlán Complex. Finally, I discuss the implications of these results for the subduction and exhumation rates of the Acatlán Complex during the Paleozoic as well as for eclogite thermobarometry.

## Geological context

The Acatlán Complex, southern Mexico, is the largest exposure (~ 10,500 km^2^) of Paleozoic metamorphic rocks in Mexico, and one of the two localities in the country where eclogites and eclogite-facies rocks have been described so far^5,15^.

The Acatlán Complex is a fault-bounded crystalline basement, bounded to the north by the Trans-Mexican Volcanic belt (Cenozoic), to the east by the Oaxacan Complex (Mesoproterozoic), to the south by the Xolapa Complex (Mesozoic), and to the by with the Guerrero-Morelos platform (Mesozoic)^15–18^ (Fig. 1). Since the work of Ortega-Gutiérrez^15^, the tectonothermal evolution and stratigraphy of the polymetamorphic Acatlán Complex have been the subject of debate^5,19–22^. Complications arise as the Acatlán Complex may record distinct orogenic cycles associated with opening and closure of the Iapetus, Rheic and Paleo-Pacific oceans^20–23^ leading to different subdivisions of the Complex, obscuring our understanding of its evolution.

This study focuses on the Piaxtla Suite (Fig. 1)—the HP belt of the Acatlán Complex where eclogite-facies metamorphism has been described. Details of the subdivision of the Acatlán Complex are described elsewhere^5,19–22^. The Piaxtla Suite is a N–S trending HP belt comprised of metasediments, metabasites, metagranitoids, and serpentinized ultramafic bodies, which record variable metamorphic grade^5,12,15,23,24^ (Fig. 1). Different areas within the Piaxtla Suite record peak blueschist- to eclogite-facies conditions, and exhumation through the amphibolite and greenschist facies^5–8,10,11,15^.

## Acatlán eclogites

Eclogites sensu lato (the word eclogite here refers broadly to variably retrogressed mafic rocks with garnet, omphacitic clinopyroxene, and rutile) in the Piaxtla Suite crops out near the towns of Mimilulco, Las Minas, Santa Cruz Organal-San Francisco de Asís, and Piaxtla (Fig. 1). Eclogites display a peak mineral assemblage of Grt + Omp + Rt ± Ph ± Czo/Zo ± Qz ± Amp^6–10,14^; this assemblage is replaced by amphibolite- (Amp + Pl + Ttn ± Czo/Zo) and greenschist-facies (Amp ± Chl) retrograde assemblages during exhumation^6–10,14^. Existing *P*–*T* conditions calculated for eclogites (including variably retrogressed samples) vary depending on the area within the Piaxtla Suite. Previous works suggest *P*–*T* conditions of 1.1–1.5 GPa and 560 ± 60 °C in the area of Mimilulco^8^. In the Santa Cruz Organal area, eclogites record 1.5–1.7 GPa and 768–830 °C^10^, and in the Piaxtla area *P*–*T* conditions of 1.1–1.3 GPa and 491–609 °C^7,8,10^. A new eclogite locality was reported by Hernández-Uribe et al.^6^ west of Piaxtla, and considered to record peak metamorphic conditions of ~ 2.2 GPa and ~ 690 °C. Other authors have reported “eclogite-facies” *P*–*T* conditions in amphibolites^9,11^, that lack omphacitic clinopyroxene, garnet, and/or rutile, and in non-mafic lithologies such as rutile-bearing mica schists^12^ and HP granitoids^5,23^.

Lu–Hf garnet–whole-rock geochronology from an amphibolitized eclogites in the Piaxtla area indicates that a single eclogite-facies metamorphism of the suite took place *c.* 351–353 Ma^14^, consistent with: (a) a Sm–Nd garnet–whole rock age of *c.* 388 ± 44 Ma^25^ (sample near town of Xayacatlán); (b) U–Pb zircon ages in retrogressed eclogites and amphibolites (interpreted as former eclogites) in the Piaxtla^26^ and San Francisco de Asís areas^9^; and (c) with amphibole ^40^Ar/^39^Ar ages of *c.* 342–344 from an amphibolite (“retrogressed eclogite” from the area of Piaxtla)^13^ and of *c.*336 ± 6 Ma of an eclogite from the Piaxtla area^10^. Other studies, however, suggest that the Acatlán Complex records more than one eclogite facies event^10,19,22^. An amphibole ^40^Ar/^39^Ar age of *c.* 430 ± 5 Ma reported from a retrogressed eclogite in the Santa Cruz Organal area was interpreted to date eclogite-facies metamorphism, followed by a *c.* 374 Ma ^40^Ar/^39^Ar phengite age in the same sample, interpreted to date cooling during exhumation^10^; however, the interpretation of the ^40^Ar/^39^Ar ages is disputed, as the samples display complex Ar spectra suggesting Ar excess or inheritance^14^.

## Thermobarometry of Acatlán eclogites

To re-explore the eclogite-facies *P*–*T* conditions of the Piaxtla Suite in the Acatlán Complex, I applied thermobarometric methods suitable for eclogite-facies mafic rocks. There are four published studies (to my knowledge) with available compositions of garnet, omphacitic clinopyroxene, and phengite in the Piaxtla Suite, which result in three eclogite localities in the Acatlán Complex (Fig. 1). These include the Piaxtla area with samples 152^7^, MP3^8^, ACA7^10^, ACA8^10^, and EC-1^6^. Towards the north, localities include Mimilulco, with sample MI6^8^ and Santa Cruz Organal, with eclogite RAC148^10^. The study of Middleton et al.^9^, from the San Francisco de Asís area (near Santa Cruz Organal; Fig. 1), was not included here because the published clinopyroxene (inclusion in amphibole) composition is a non-omphacitic clinopyroxene.

**Figure 2:**
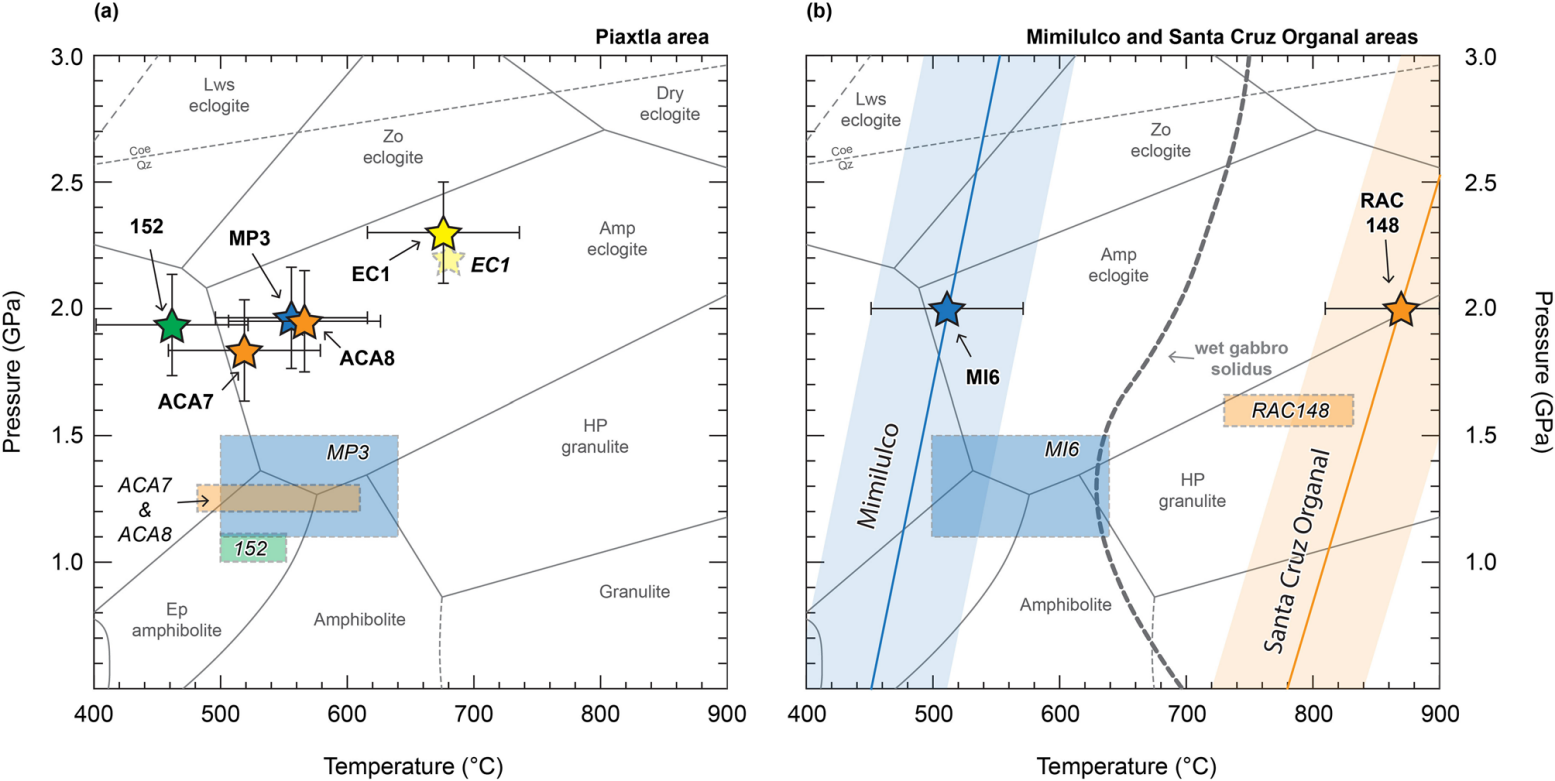
Pressure and temperature (*P*–*T*) conditions of Piaxtla Suite eclogites. (**a**) Piaxtla area and (**b**) Mimilulco and Santa Cruz Organal areas. The colored stars represent the mean *P*–*T* conditions and the error bars indicate the ± 0.2 GPa and ± 60 °C uncertainty associated to the thermobarometric methods (see Methods). The shaded colored rectangles with the sample number in italics correspond to the previously calculated *P*–*T* conditions for the same samples used in this study. The colors of the stars and boxes refer to: 152 (green)—Ortega-Gutiérrez^7^; MP3 and MI6 (blue)—Meza-Figueroa et al.^8^; ACA7, ACA8, and RAC148 (orange)—Vega-Granillo et al.^10^; EC-1 (yellow)—Hernández-Uribe et al.^6^.

Pressures were calculated using the garnet–clinopyroxene–phengite barometer of Ravna and Terry^27^. Temperatures were obtained using the garnet–clinopyroxene thermometer calibrations of Ravna^28^, Nakamura^29^, and Sudholz et al.^30^. While some of these thermobarometers have been available for several decades, their application for the Acatlán eclogites is still novel. For internal consistency, the reported results in the text and in Fig. 2 correspond to the calculated mean of the intersections between the Ravna and Terry^27^ barometer and Ravna^28^ thermometer (Table 1). Temperatures obtained from the Nakamura^29^ and Sudholz et al.^30^ calibrations are also given in Table 1. Details of the methodology and related uncertainties are provided in the Methods section.

**Table 1:** Calculated *P⎼T* conditions of Acatlán eclogites.

	P (GPa)^a^					T (°C)^b^					T (°C)^c^				T (°C)^d^			
	*n*	Max	Min	Mean	2σ	*n*	Max	Min	Mean	2σ	Max	Min	Mean	2σ	Max	Min	Mean	2σ
**Piaxtla area**																		
152	4	2.07	1.82	1.94	0.199	4	509	430	468	65	483	446	457	35	509	435	471	60
MP3	8	2.04	1.9	1.97	0.092	8	571	539	555	24	528	510	520	14	576	549	562	23
ACA7	8	1.88	1.79	1.83	0.077	8	525	512	519	11	547	529	536	18	600	571	586	28
ACA8	8	2.01	1.87	1.94	0.093	8	574	556	565	13	584	566	576	18	626	611	619	12
EC1	4	2.33	2.28	2.30	0.004	4	680	670	675	12	646	640	643	7	691	684	688	7
**Mimilulco and Santa Cruz Organal areas**																		
MI6*	–	–	–	–	–	1	–	–	511	–	–	–	498	–	–	–	529	–
RAC148*	–	–	–	–	–	4	890	850	870	35	832	805	818	23	907	882	895	35

*Temperatures calculated at 2.0 GPa.

^a^Ravna and Terry^27^; ^b^Ravna^28^; ^c^Nakamura^29^; ^d^Sudholz et al.^30^.

Different eclogites from the Piaxtla area yield similar *P*–*T* conditions (Fig. 2a). Eclogite MP3 yields *P*–*T* conditions of 1.97 GPa and 555 °C, eclogites ACA7 and ACA8 yields conditions of 1.83 GPa and 519 °C and 1.94 GPa and 565 °C, respectively. Eclogite 152 yields *P*–*T* conditions of 1.94 GPa and 468 °C (Fig. 2a). Eclogite EC-1, from the new locality west to the area of Piaxtla, yields 2.3 GPa and 675 °C (Fig. 2a), the greatest of all the Acatlán Complex.

In contrast to the Piaxtla eclogites, the Mimilulco and Santa Cruz Organal eclogites yield contrasting temperature estimates (Fig. 2b); unfortunately, there are no phengite analyses from either localities, thus no new pressure estimates were calculated. The Mimilulco eclogite MI6 yields a garnet–clinopyroxene temperature of 511 °C at 2.0 GPa (only one garnet–clinopyroxene pair available^8^). By contrast, the Santa Cruz Organal eclogite RAC148 yields a temperature of 870 °C at 2.0 GPa (Fig. 2b).

## Discussion

### Comparison with previous studies

Temperatures calculated in this work are similar to previously published estimates for eclogites in different parts of the Acatlán Complex^6–8,10^ (Fig. 2). However, our barometric calculations show substantially—and systematically—higher pressures than previously calculated across the Acatlán Complex (Fig. 2). For instance, in the Piaxtla area, the calculated pressure is ~ 2.0 GPa for four different samples (eclogite 152, MP3 ACA7, and ACA8; Fig. 2) whereas previous studies^7,8,10^ suggested that the eclogite-facies metamorphic event in this area occurred at ~ 1.1–1.5 GPa*;* such estimates are different even when considering the ± 0.2 GPa uncertainty related to the barometer (see Methods).

Unfortunately, the lack of phengite in the eclogites from the Mimilulco and Santa Cruz Organal areas precluded the recalculation of new pressures with the approach used in this work. Yet, there is no reason for why the 1.1–1.5 GPa for the Mimilulco eclogite and 1.5–1.7 GPa for the Santa Cruz Organal eclogite could not be higher than previously reported. For example, Meza-Figueroa et al.^8^ suggest that the Mimilulco eclogite (MI6) was metamorphosed at the same *P–T* conditions than eclogite MP3 from Piaxtla^8^; thus the Mimilulco eclogite could also record pressures of ~ 2.0 GPa. By contrast, it is more challenging to infer a pressure for the Santa Cruz Organal eclogite, as *P*–*T* conditions are not available for other eclogites near the area. Previous work in the San Francisco de Asís area (relatively near Santa Cruz Organal; Fig. 1) estimated pressures > 1.6 GPa^9^, similar to that calculated by Vega-Granillo et al.^10^ for the Santa Cruz Organal eclogite (1.5–1.7 GPa), but with contrasting temperatures (650–750 °C^9^ vs 768–830 °C^10^). The temperature mismatch between these studies may be explained either by the fact that eclogites in both localities experienced different *P*–*T* conditions or due to the use of non-omphacitic clinopyroxene for the thermobarometry^9^. The fact that the Santa Cruz Organal eclogite records the highest temperature in the Acatlán Complex (Fig. 2 and Table 1) may suggest that the pressure could be different to other eclogites in the Piaxtla Suite as well.

A recent petrologic study combining phase-equilibrium modeling and Zr-in-rutile thermometry for an eclogite from a new locality west of Piaxtla area obtained conditions of ~ 2.2 GPa and ~ 690 °C^6^. Our thermobarometric calculations for the same eclogite sample (EC-1) yield similar *P–T* conditions than previously calculated (Fig. 2; Table 1). Importantly, the agreement between the previous *P–T* calculations from Hernández-Uribe et al.^6^ with the conditions obtained here, further support our findings for the other eclogites in different parts of the Acatlán Complex.

### Implications for the geodynamic evolution

The difference between the previously reported pressures for all the complex and the contrastingly higher pressure in the new eclogite locality was interpreted to be an artifact due to differences in thermobarometric methods^6^. However, here, I obtained similar pressures from different parts of the Piaxtla Suite of the Acatlán Complex using conventional thermobarometric approaches (Fig. 2). Therefore, I argue that these new results indicate systematic deeper subduction than previously thought. If no errors are considered, the calculated pressures of ~ 1.9–2.3 GPa, and corresponding inferred depths (63–75 km; see methods for pressure-to-depth conversion) suggest that different areas within the complex record slightly different depths. On the other hand, if the uncertainties in the calculations are considered (± 0.2 GPa, ± 6–7 km), then the calculated pressures and inferred depths in this work converge suggesting the complex reached a similar depth during subduction.

Temperatures from the Piaxtla and Mimilulco areas are the same considering the ± 60–100 °C uncertainty related to the thermometric calculations. However, the temperature calculated here and in a previous work^10^ for the Santa Cruz Organal eclogite indicate that this area records the highest temperature of any rocks in the Acatlán Complex (Fig. 2). The differences in calculated temperatures could suggest different locations of the proto-Acatlán Complex within the subducting slab (i.e., hotter towards the slab top vs colder towards the bottom). Regardless of the temperature interpretation, the greatest depths obtained here situates the subducting proto-Acatlán Complex deeper than previously hypothesizes by all the tectonic models for the region.

The greater pressures–depths calculated here for the eclogites imply a faster subduction–exhumation cycle for the Acatlán Complex. Simple tectonic-rate calculations for the Acatlán Complex were obtained by using: (a) the youngest depositional ages of the sediments above the mafic oceanic crust; (b) the greatest depth reached during subduction and related eclogite-facies age (considering a single event); as well as (c) the depth and time of exhumation. For calculating the burial rate, I use the youngest detrital zircon in a metapsammite in the Piaxtla Suite with an age of *c.* 365 Ma interpreted to represent the youngest depositional limit^13^. Coupled with the depth from this work (i.e. 75 km) and an eclogite-facies age of *c.* 353 Ma (Lu–Hf garnet–whole-rock^13^), I obtained a linear burial rate of ~ 6.3 mm/yr, well within the estimates of convergence rates of tectonic plates in subduction zones^31^. Furthermore, the eclogite-facies data obtained here combined with muscovite ^40^Ar/^39^Ar cooling age of *c.*334 Ma in a retrogressed eclogite^13^ with amphibolite-facies *P*–*T* conditions of ~ 0.6 GPa^8^ equates to an exhumation rate of ~ 2.8 mm/yr, similar to other HP terranes worldwide^32^. In summary, these simple calculations indicate it took the proto-Acatlán Complex ~ 12 Myr to subduct to ~ 75 km depth, and ~ 19 Myr to return to crustal depths, resulting in a subduction–exhumation cycle of ~ 31 Myr. These calculations contrast and thus challenge models for the Acatlán Complex with slower subduction and exhumation rates. For example, a previous burial rate of 2.7 mm/yr^13^ and an exhumation rate of 2.4 mm/yr^13^ are 3.6 mm/yr and 0.8 mm/yr slower, respectively, than the ones calculated here. The discrepancy between the calculated burial–exhumation cycle may be explained by the input data, as the burial and exhumation rates are strongly dependent in the timing of both the formation of the eclogite protolith and the exhumation to crustal depths. However, regardless of these data, the thermobarometric and new depth calculations obtained in this work would unequivocally result in faster tectonic rates.

### Implications for eclogite thermobarometry

The results presented here indicate that for the Acatlán eclogites, conventional thermobarometric methods, phase-equilibrium modeling^6^, and Zr-in-rutile thermometry^6^ yield consistent *P–T* conditions (Fig. 2a). While the uncertainties related to conventional thermobarometric methods (see Methods section) are considerably larger than the ones from other methods^33^, I argue that in relatively well-equilibrated rocks, *P–T* estimates should be similar. Thus, as shown by other studies^2,34,35^, the obtention of reliable *P–T* data needs to involve the application of different thermobarometric methods.

The temperatures obtained using different calibrations are the same including uncertainties (Table 1). The calibration from Sudhloz et al.^30^ yields the highest temperatures compared to the other calibrations, whereas the Nakamura^29^ calibration tend to yield the lowest temperature of all the thermometers (Table 1). Importantly, the Sudhloz et al.^30^ calibration was parametrized from high-temperature experiments of mantle lithologies, potentially explaining why such temperatures are the highest. Yet, the mineral compositions from Acatlán eclogites are within the ranges recommended for that calibration (Supplementary Tables S1–S3). Further, from all the calibrations used here, only the Sudhloz et al.^30^ thermometer includes a correction for the jadeite content in clinopyroxene, which is key for yielding reliable temperatures for subduction-related eclogites^36,37^.

The comparison between the calculated temperatures in this work and the study of Hernández-Uribe et al.^6^ (which used Zr-in-rutile thermometry) indicates that the Sudhloz et al.^30^ thermometer yields almost identical temperatures than the Zr-in-rutile thermometer (695 °C^6^ vs 688 °C). By contrast, such Zr-in-rutile temperatures differ the most from the Nakamura^29^ temperature (695 °C^6^ vs 643 °C). Therefore, the comparison between independent approaches seems to suggest that the Sudhloz et al.^30^ calibration may yield more reliable temperatures for subduction-related eclogites than the other Fe–Mg garnet–clinopyroxene thermometers.

## Methods

Barometric calculations were done using the garnet–clinopyroxene–phengite barometer with the calibration of Ravna and Terry^27^. The garnet–clinopyroxene–phengite barometer relies in the net transfer reaction between garnet, clinopyroxene, and phengite (mineral abbreviations follow Warr^38^):

1 $$ 6Di + 3Ms = 2Grs + 1Prp + 3Cel $$

where the equilibrium constant (*K*_1_) of this reaction can be expressed as:

2 $$ K_{1} = \frac{{(a_{Prp}^{Grt} )(a_{Grs}^{Grt} )^{2} (a_{Cel}^{Ph} )^{3} }}{{(a_{Di}^{Cpx} )^{6} (a_{Ms}^{Ph} )^{3} }} $$

Temperatures were calculated using the garnet–clinopyroxene thermometer using the Ravna^28^, Nakamura^29^, and Sudholz et al.^30^ calibrations. The garnet–clinopyroxene thermometer relies on the exchange of Fe^2+^ and Mg between garnet and clinopyroxene. The equilibrium Fe^2+^–Mg distribution coefficient (*K*_D_) can be expressed as:

3 $$ K_{D} = \frac{{\left( {\frac{{Fe^{2 + } }}{Mg}} \right)^{Grt} }}{{\left( {\frac{{Fe^{2 + } }}{Mg}} \right)^{Cpx} }} $$

Uncertainties related to the conventional thermobarometers applied here are commonly quoted to be ± 0.2 GPa for the barometer^27,39,40^ and ± 60 °C for the thermometer^27,28,37^. For the latter, temperatures can be up ± 100 °C due to the Fe^3+^ estimation in clinopyroxene^41^. In this work, Fe^3+^ in clinopyroxene calculated with the following relation: Fe^3+^ = Na–Al–Cr. This Fe^3+^ recalculation scheme was used instead of the stochiometric Fe^3+^ as the latter resulted in unrealistic lower garnet–clinopyroxene temperatures (< 350 °C).

For the *P*–*T* calculations, published garnet, clinopyroxene, and phengite chemical compositions^6–8,10^ were used from eclogites distributed along different portions of the Piaxtla Suite within the Acatlán Complex (Fig. 1; Supplementary Tables S1–S3). When provided, available petrological context in the publications (e.g., rims vs core and/or interpretations of peak vs retrograde), were considered for the *P*–*T* calculations. For samples MP3^8^, MI6^8^, and EC-1^6^, all the mineralogical data come from such papers. Data for eclogite 152^7^ was partially published by Ortega-Gutiérrez^7^; the complete analyses were kindly provided by the author. Similarly, data for ACA7, ACA8, and RAC148 was partially published by Vega-Granillo et al.^10^. Complete analyses were obtained from that author’s doctoral dissertation. In this case, we only picked a pair of each mineral for the *P*–*T* calculations. All mineralogical analyses used for the thermobarometric calculations are given in Tables S1–S3 in the Supplementary Material.

Pressure-to-depth conversion uses a layered model assuming a total crustal thickness of 30 km, where the upper crust is 20 km and has a density of 2.8 g/cm^3^, and where the lower crust is 10 km and has a density of 2.9 g/cm^3^. The crust is followed by an upper mantle with density of 3.3 g/cm^3^. A greater crustal thickness results in greater subduction depths, whereas changes in the considered densities would have minor effects on the overall calculated depth. Tectonic overpressure was not considered in our pressure-to-depth calculations but deviation from lithostatic pressure is likely within the order of the depth uncertainty related to the barometric method used here^42^.

## Supplementary Information

Supplementary Information.

## Data Availability

All data used for the thermobarometric calculations are provided in Supplementary Information. The excel spreadsheet used the calculations is available at https://doi.org/10.1111/j.1525-1314.2004.00534.x.
